# Pre-existing Type 2 Diabetes Mellitus Is an Independent Risk Factor for Mortality and Progression in Patients With Renal Cell Carcinoma

**DOI:** 10.1097/MD.0000000000000183

**Published:** 2014-12-12

**Authors:** Antonio Vavallo, Simona Simone, Giuseppe Lucarelli, Monica Rutigliano, Vanessa Galleggiante, Giuseppe Grandaliano, Loreto Gesualdo, Marcello Campagna, Marica Cariello, Elena Ranieri, Giovanni Pertosa, Gaetano Lastilla, Francesco Paolo Selvaggi, Pasquale Ditonno, Michele Battaglia

**Affiliations:** From the Department of Emergency and Organ Transplantation, Urology, Andrology and Kidney Transplantation Unit, University of Bari, Bari (AV, GL, MR, VG, MC, PS, PD, MB); Department of Medical and Surgical Sciences, Nephrology, Dialysis and Transplantation Unit, University of Foggia, Foggia (GG, ER); Department of Emergency and Organ Transplantation – Nephrology, Dialysis and Transplantation Unit (SS, MC, GP); and Department of Pathology, University of Bari, Bari, Italy (GL).

## Abstract

Malignancies are one of the main causes of mortality in diabetic patients; however, to date, very limited data have been reported on the specific influence of type 2 diabetes mellitus (T2DM) on the survival of patients with renal cell carcinoma (RCC). In the present long-term retrospective study, we investigated whether T2DM may influence the overall survival (OS), cancer-specific survival (CSS), and progression-free survival (PFS) in patients with surgically treated RCC.

Medical records of 924 patients treated by radical or partial nephrectomy for sporadic, unilateral RCC were reviewed. Patients with type-1 DM and with T2 DM receiving insulin treatment were excluded. Survival estimates were calculated according to the Kaplan–Meier method and compared with the log-rank test. Univariate and multivariate analyses were performed using the Cox regression model.

Of the 924 RCC patients, 152 (16.5%) had T2DM. Mean follow-up was 68.5 months. Mean OS was 41.3 and 96.3 months in T2DM and non-T2DM patients, respectively (*P* < 0.0001).

The estimated CSS rates at 1, 3, and 5 years in T2DM versus non-T2DM patients were 63.4% versus 76.7%, 30.4% versus 56.6%, and 16.3% versus 48.6%, respectively (*P* = 0.001). Mean PFS was significantly lower (31.5 vs 96.3 months; *P* < 0.0001) in the T2DM group. At multivariate analysis, T2DM was an independent adverse prognostic factor for OS (hazard ratio [HR] = 3.44; 95% confidence interval [CI]:2.40–4.92), CSS (HR = 6.39; 95% CI: 3.78–10.79), and PFS (HR = 4.71; 95% CI: 3.11–7.15).

In conclusion, our findings suggest that patients with RCC and pre-existing T2DM have a shorter OS, increased risk of recurrence, and higher risk for kidney cancer mortality than those without diabetes.

## INTRODUCTION

According to the 2014 National Diabetes Statistics Report, type 2 diabetes mellitus (T2DM) is the most common form of diabetes, affecting 90% to 95% of the 29.1 million Americans with this disease.^1^

Even though cardiovascular complications remain the major cause of morbidity and mortality in diabetic patients, epidemiological studies indicate that these patients have an increased risk of several types of cancer.^2–5^ About 40% of the years of life lost due to diabetes can be attributed to nonvascular conditions, including about 10% attributable to death from cancer.^6^ Together with the increasing incidence of DM, the incidence of renal cell carcinoma (RCC) has also been increasing worldwide over the past decades. It is estimated that in 2014, 63,920 new cases will be diagnosed and 13,860 patients will die of RCC in the United States.^7^ Although many proteins have been investigated as prognostic factors, no clinically useful circulating marker is yet available for RCC.^8,9^

Data about the relationship between DM (in particular T2DM) and RCC are currently controversial. Nevertheless, a recent meta-analysis suggests that DM is associated with a 42% increased risk of kidney cancer.^10^ Although it is clear that malignancies are one of the main causes of mortality in diabetic patients,^6^ very limited data have yet been reported on the specific influence of T2DM on the survival of patients with renal cancer. Therefore, in the present long-term retrospective study, we investigated whether T2DM may influence the overall survival (OS), cancer-specific survival (CSS), and progression-free survival (PFS) in patients with surgically treated RCC.

## METHODS

### Study Population

We reviewed medical records of 924 patients treated by radical or partial nephrectomy for sporadic, unilateral RCC from 1979 to 2013 at the Urology Unit, Department of Emergency and Organ Transplantation of University of Bari (Italy). The study was approved by the local ethics committee and performed in accordance with the ethical standards laid down in the 1964 Declaration of Helsinki and later amendments. Written informed consents were obtained from all participants after full explanation of the purpose and nature of all procedures used. The following demographic and clinical-pathologic features were analyzed: age, sex, hypertension, body mass index (BMI), DM diagnosis, tumor size, pathological tumor-node-metastasis (pTNM) stage, histological subtype, tumor necrosis, and Fuhrman nuclear grade. Tumor staging was reassessed according to the 2009 American Joint Committee on Cancer/Union International Contre le Cancer TNM classification. The Fuhrman grading system and Heidelberg histological classification were used to define the tumor grade and histological subtype. Patients without radiographic or palpable evidence of lymphadenopathy generally did not undergo lymphadenectomy (Nx) and were grouped with pathologic N0 patients for analysis. Tumor size was recorded as the largest diameter (cm) described in the pathology report. Tumor necrosis was recorded as present/absent. Regressive changes (fibrosis, hyalinization, or cystic transformation) were not considered as necrosis. The TNM classification, Fuhrman nuclear grade, and tumor necrosis were assigned by blinded re-review of all surgical specimens by a single urologic pathologist. All patients were preoperatively staged by thoracoabdominal computed tomography (CT) or magnetic resonance imaging, as recommended by the European Association of Urology (EAU) guidelines. The study included RCC patients with T2DM lasting at least 3 years, and RCC patients with normal fasting plasma glucose values (non-T2DM patients), according to their preoperative medical records. Patients were followed-up for blood glucose levels, and in non-T2DM group, the levels remained in the normal range. As some diabetic patients may have normal fasting blood glucose values, laboratory analysis was repeated 2 times before surgery. In doubtful cases, an endocrinological evaluation for more specific tests was performed and patients with impaired fasting glucose or impaired glucose tolerance were excluded from the study. Patients with type-1 DM and with T2DM receiving insulin treatment (including T2DM patients that needed insulin treatment for uncontrolled blood glucose levels during the follow-up period) were excluded. Follow-up information was obtained from office visits or telephone interviews. Follow-up time was calculated in months from the date of surgery to the last medical checkup or death. Patients were assessed by CT or ultrasonography and/or chest X-rays, as recommended by the EAU guidelines, to evaluate tumor recurrence. CSS and OS, with a minimum follow-up period of 4 months, were considered.

### Statistical Analysis

Data are expressed as mean ± standard deviation. MedCalc 9.2.0.1 (MedCalc software, Mariakerke, Belgium) and PASW 18 software (PASW 18, SPSS Inc, Chicago, IL) were used for statistical analyses. OS was defined as the time from surgery to death or June 2014 for living patients. In the CSS analysis, patients still alive or lost to follow-up were censored, as well as patients who died from RCC-unrelated causes. PFS was determined from the date of surgery to the date of relapse. Survival estimates were calculated according to the Kaplan–Meier method and compared with the log-rank test. Univariate and multivariate analyses were performed using the Cox proportional hazards regression model to identify the most significant variables for predicting OS, CSS, and PFS. Only the variables that were statistically significant at univariate analysis were used for the Cox regression model. The backward selection procedure with removal criterion *P* > 0.10 based on likelihood ratio tests was performed. A *P* value <0.05 was set as statistically significant.

## RESULTS

This retrospective study included 924 consecutive RCC patients, 196 (21%) of whom underwent partial and 728 (79%) radical nephrectomy. The main clinical characteristics are summarized in Table 1. Pre-existing T2DM was present in 152 RCC patients (16.5%); 104 of 152 (68%) patients were hypertensive. The overall population was followed up for a mean period of 68.5 months (range: 4–326). Mean age of T2DM patients was significantly higher (66 vs 59 years, *P* < 0.001), as well as the percentage of hypertension (68% vs 45%, *P* < 0.001) and mean BMI (30.3 vs 27.8, *P* < 0.001). No significant difference in renal function was observed between T2DM and non-T2DM patients (69.0 ± 21.9 vs 77.1 ± 21.82 mL/min/1.73m^2^, respectively, *P* = 0.6).

**TABLE 1:**
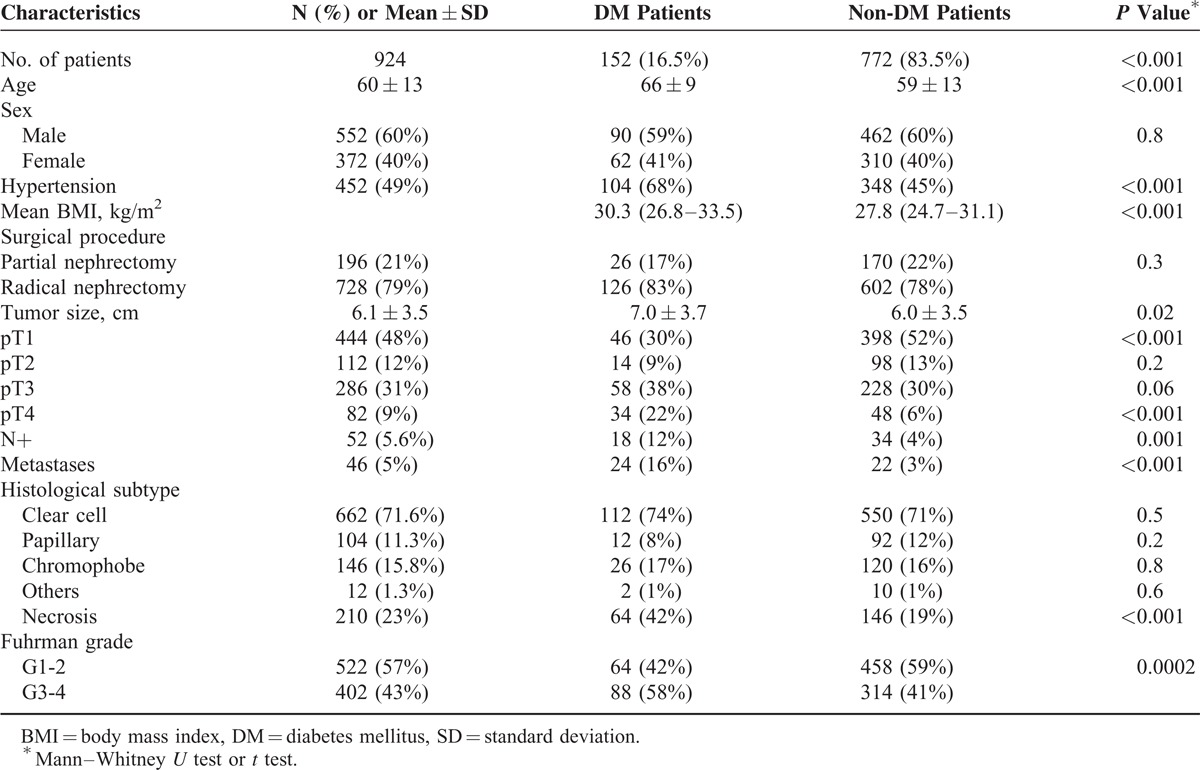
Patients’ Clinical and Pathological Characteristics

At the time of surgery, 52 patients (5.6%) had lymph node involvement (12% T2DM, 4% non-T2DM, *P* = 0.03) and 46 patients (5.0%) had metastatic disease (16% T2DM, 3% non-T2DM, *P* < 0.001). T2DM patients showed a larger tumor size (*P* = 0.02) and a higher percentage of tumor necrosis (*P* < 0.001) as compared with non-T2DM patients.

**FIGURE 1:**
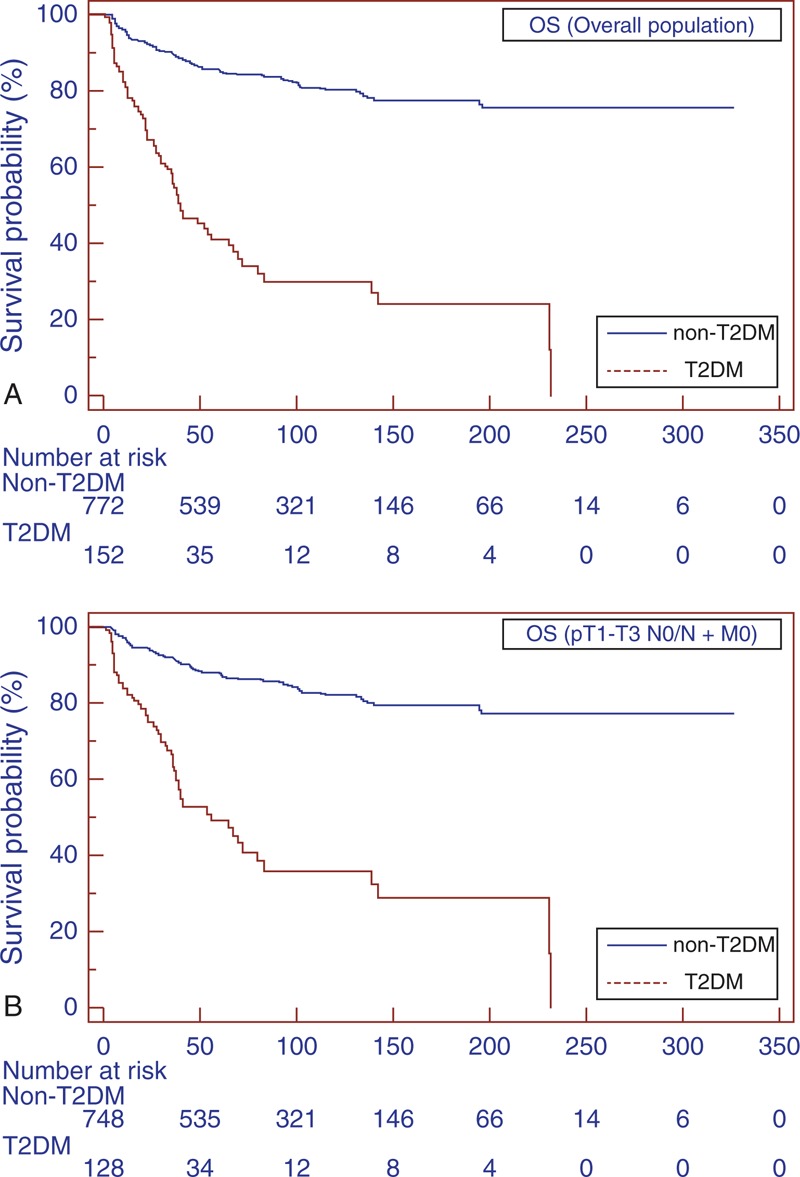
Kaplan–Meier OS curves, stratified by T2DM and non-T2DM groups for the overall population (A). Kaplan–Meier OS curves for localized RCC (B). OS = overall survival, RCC = renal cell carcinoma, T2DM = type-2 diabetes mellitus.

Mean OS was 41.3 and 96.3 months in T2DM and non-T2DM patients, respectively (*P* < 0.0001) (Figure 1A); 76 patients died of nonrelated RCC causes. At multivariate analysis, advanced age, high BMI, the presence of T2DM, tumor size, pathological stage, and presence of distant metastases were independent adverse prognostic factors for OS (Table 2).

**TABLE 2:**
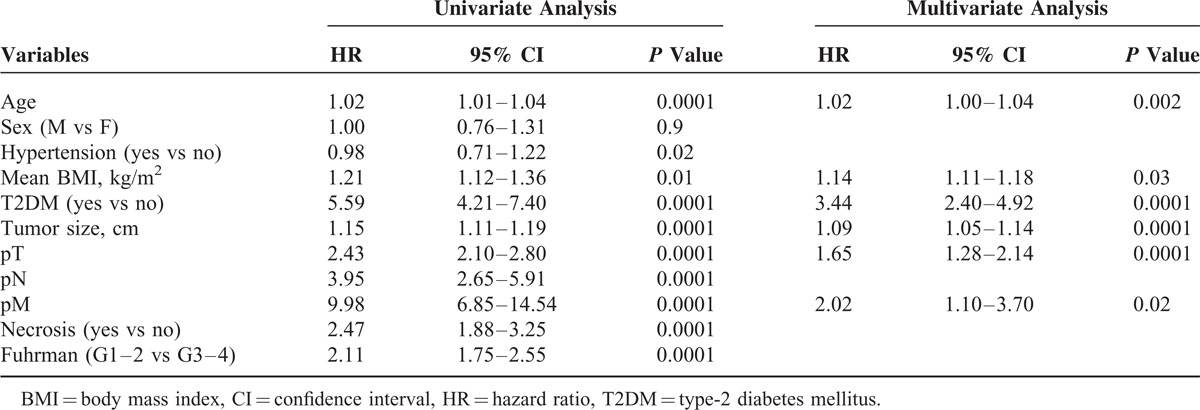
Univariate and Multivariate Analyses (Cox model) for Overall Survival

**FIGURE 2:**
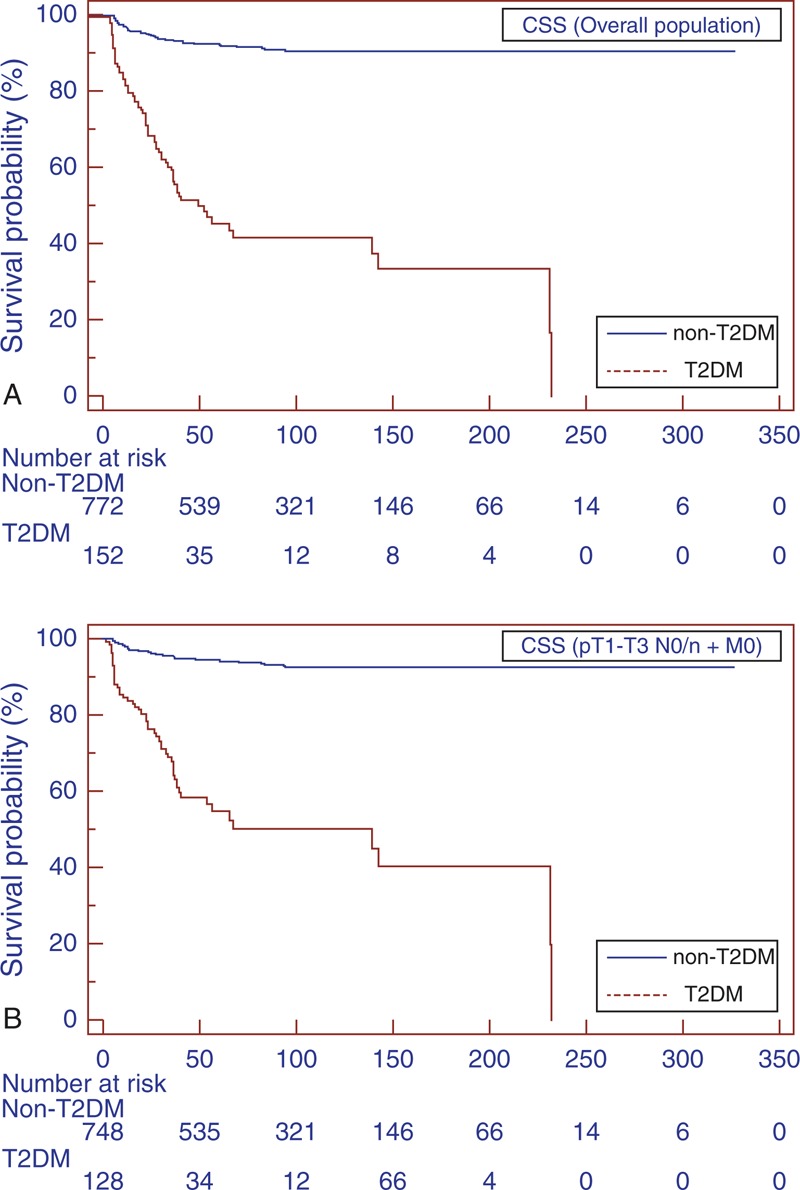
Kaplan–Meier CSS curves, stratified by T2DM and non-T2DM groups for the overall population (A). Kaplan–Meier CSS curves localized RCC (B). CSS = cancer-specific survival, RCC = renal cell carcinoma, T2DM = type-2 diabetes mellitus.

**FIGURE 3:**
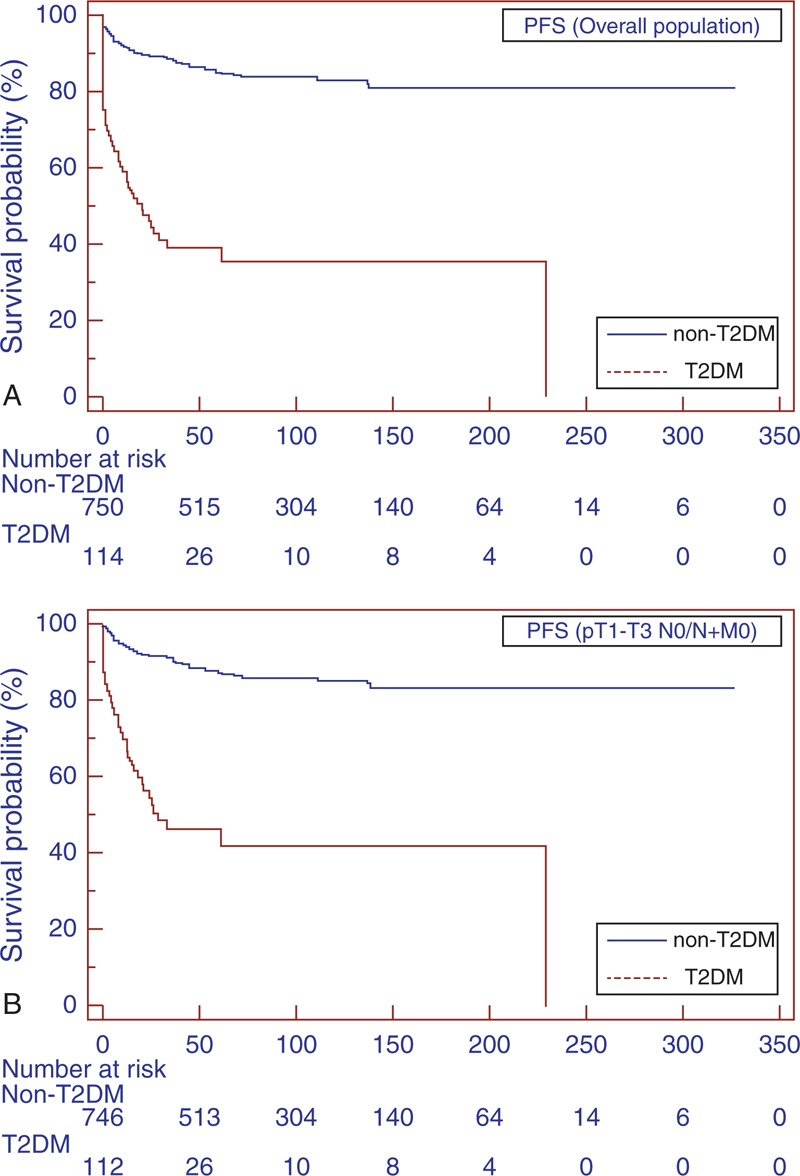
Kaplan–Meier PFS curves, stratified by T2DM and non-T2DM groups for the overall population (A). Kaplan–Meier PFS curves for localized RCC (B). PFS = progression-free survival, RCC = renal cell carcinoma, T2DM = type-2 diabetes mellitus.

The estimated CSS rates at 1, 3, and 5 years in T2DM versus non-T2DM patients were 63.4% versus 76.7%, 30.4% versus 56.6%, and 16.3% versus 48.6%, respectively (*P* = 0.001) (Figure 2A). Mean PFS was significantly lower (31.5 vs 96.3 months; *P* < 0.0001) in the T2DM group (Figure 3A).

**FIGURE 4:**
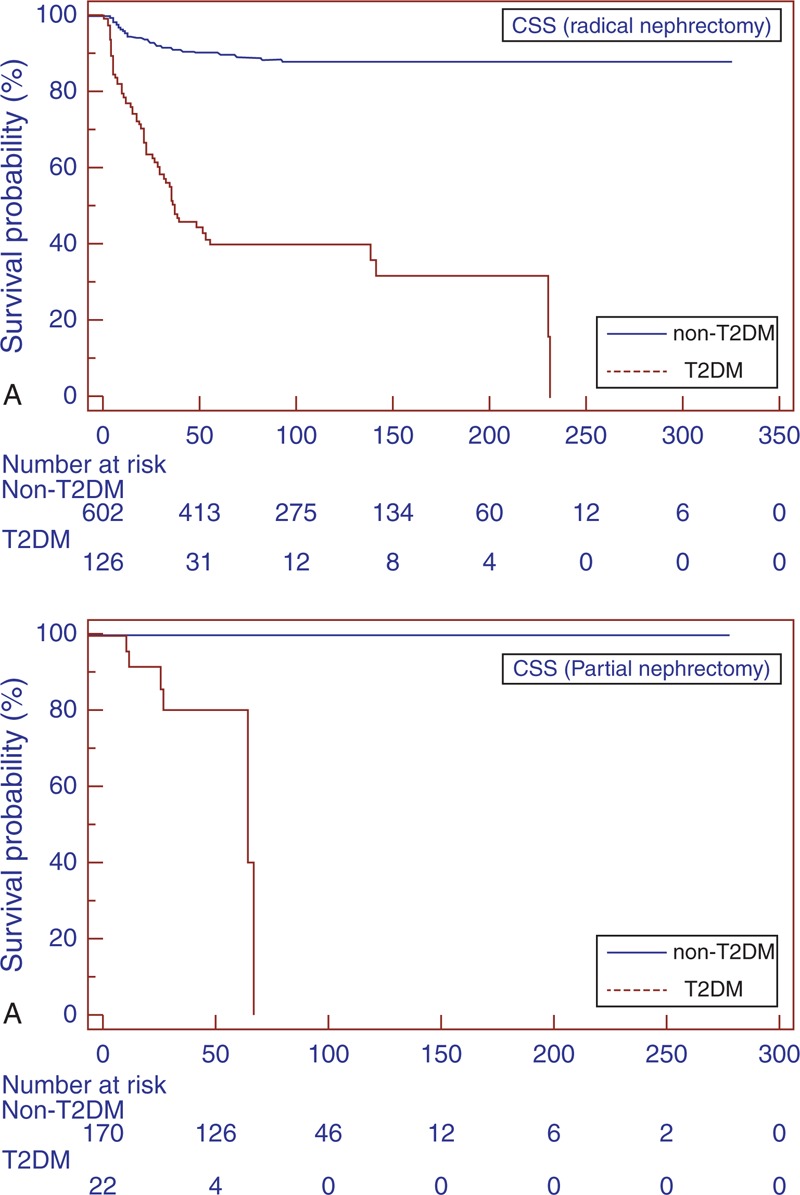
Kaplan–Meier CSS curves, stratified by T2DM and non-T2DM groups for radical (A) and partial nephrectomy (B). CSS = cancer-specific survival, T2DM = type-2 diabetes mellitus.

**FIGURE 5:**
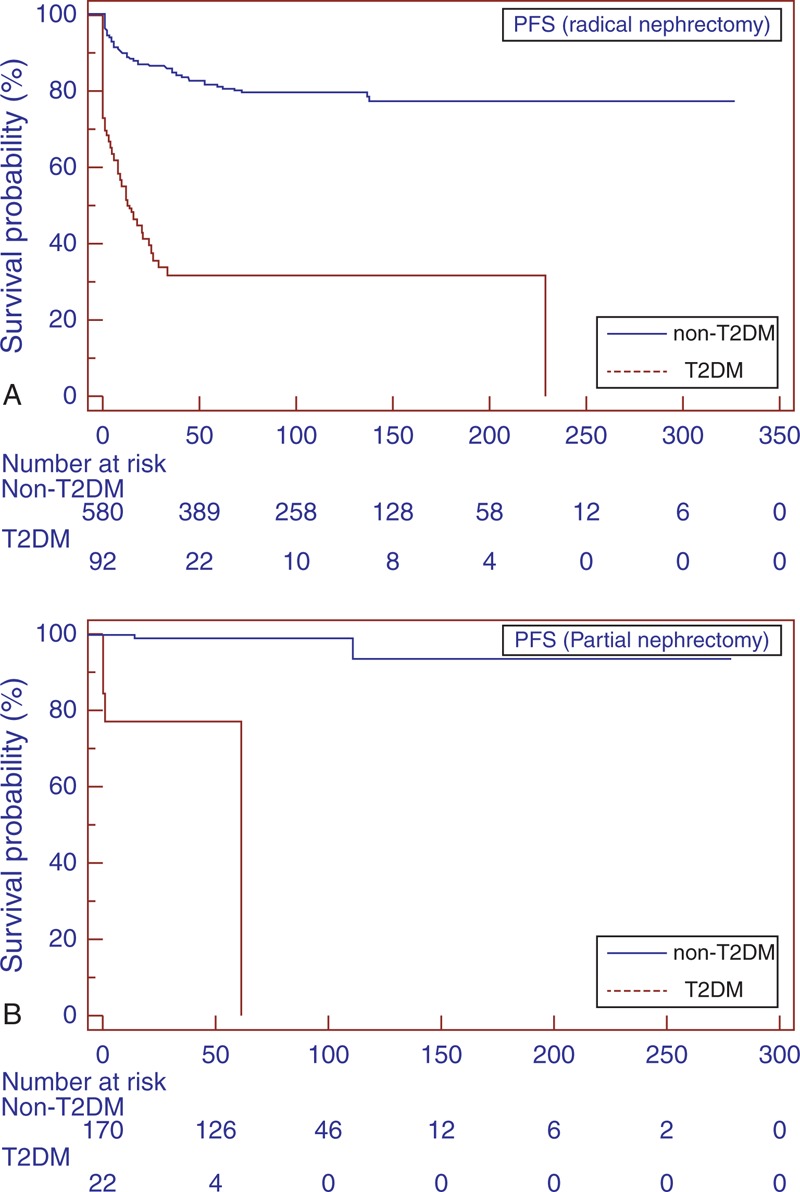
Kaplan–Meier PFS curves, stratified by T2DM and non-T2DM groups for radical (A) and partial nephrectomy (B). PFS = progression-free survival, T2DM = type-2 diabetes mellitus

Subgroup analyses for patients with localized RCC (pT1–T3 N0/N + M0) confirmed the findings in the overall population (Figure 1B, 2B, and 3B). Similar results were found when patients were stratified for type of surgery (Figures 4 and 5).

Univariate analysis for the predefined variables showed that hypertension, BMI, T2DM, tumor size, pathological stage, presence of nodal and distant metastases, tumor necrosis and Fuhrman grade were significantly associated with the risk of cancer-specific death (Table 3). At multivariate analysis the presence of T2DM, tumor size, pathological stage and presence of distant metastases were independent adverse prognostic factors for CSS (Table 3). Similar results were found for PFS (Table 4). A significant excess risk emerged for T2DM patients with an HR = 6.39 (95% CI; 3.78–10.79) for cancer-specific mortality and an HR = 4.71 (95% CI: 3.11–7.15) for progression as compared with non-T2DM patients.

**TABLE 3:**
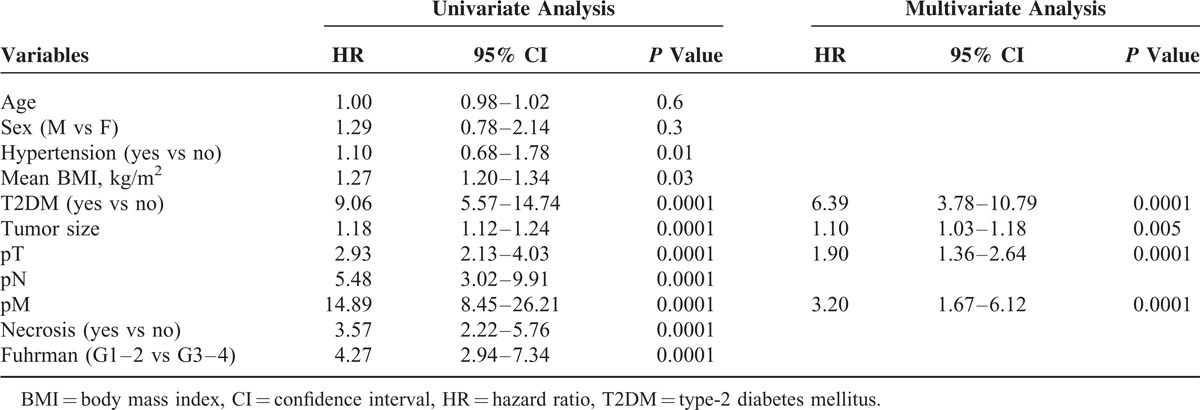
Univariate and Multivariate Analyses (Cox model) for Cancer-specific Survival

**TABLE 4:**
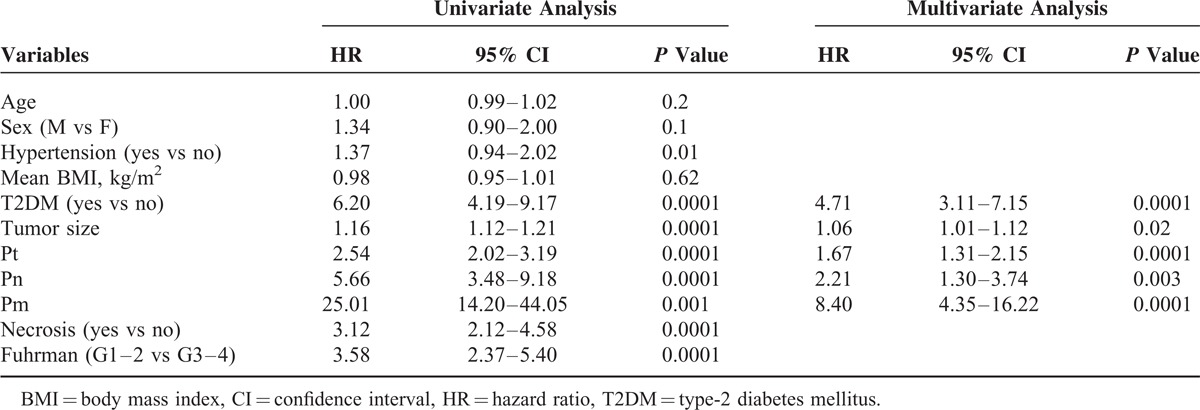
Univariate and Multivariate Analyses (Cox Model) for Progression-free Survival

## DISCUSSION

Over the last decade, many studies have investigated the association between diabetes and cancer and numerous meta-analyses have described an increased incidence of solid tumors among diabetic patients.^11–14^ DM increases the risk of renal cancer in some cohort studies,^15,16^ but it is not an established risk factor.^17^ In the current study, we found that the prevalence of T2DM within our surgical RCC population was 16.5%, which is consistent with previous reports observing that 10% to 22.5% of RCC surgical cohorts are diabetic.^18–20^ The results reported in the Barone meta-analysis that pre-existing DM in cancer patients was associated with an HR of 1.41 for the risk of all-cause mortality compared with individuals without DM,^4^ led us to reconsider the association between T2DM and kidney cancer. In the present paper, we reported a long-term retrospective single-center study showing that pre-existing T2DM in RCC patients at the time of diagnosis was associated with a higher risk of all-cause mortality compared with individuals without diabetes. This association was present not only for OS but also for renal cancer as the underlying cause of death. Therefore, T2DM may significantly reduce CSS in RCC patients. No solid, unequivocal data are available about the role of diabetes in modifying the survival of RCC patients from previous population-based studies. A single-center retrospective study conducted by Antonelli et al showed that among >1600 patients treated at their institution since 1987, 8.9% of whom were diabetic, pre-existing T2DM was not an adverse prognostic factor in patients with non-metastatic RCC.^21^ Höfner et al reported similar results in their retrospective case–control study of 1140 patients with localized RCC undergoing radical or partial nephrectomy. Although, in terms of OS, T2DM along with older age and high BMI at the time of surgery were independent risk factors for the occurrence of the event death, no significant impact on CSS and recurrence-free survival was found.^20^ In a study of a surgical series of 492 patients, T2 DM was not detected as an independent prognostic factor for RCC, and although CSS and OS were lower in the DM group, this difference was not statistically significant.^22^ Fukushima et al showed, for the first time, that DM was an independent predictor of recurrence, especially in obese patients, but not an independent risk factor of death, likely due to the small number of patients who died of RCC or any other cause, and the short follow-up.^19^ Finally, in a more recent matched cohort analysis of a series of 1964 consecutive patients with surgically treated M0 clear cell RCC, Psutka et al^23^ observed that DM was independently associated with an increased risk of both cancer-specific and all-cause mortality at multivariable analysis, adjusting for comorbidity and BMI.

In our findings, T2DM was significantly predictive of risk of cancer-specific death, and remained an independent prognosticator of outcome for PFS. Moreover, subgroup analyses for patients with localized RCC confirmed the differences in CSS and PFS, observed for the overall population.

Although several explanations have been proposed for the association between cancers and T2DM, the mechanisms underlying the influence of DM on cancer incidence and progression are still largely unknown. The American Diabetes Association and the American Cancer Society indicated that it is unclear whether such associations are direct (eg, due to hyperglycemia) or indirect (eg, due to hyperinsulinemia), or due to shared risk factors (eg, obesity), or a combination of these factors.^24–27^ Some conditions influencing the pathogenesis of T2DM, including obesity, may play an important role in cancer development and progression.^24^ In addition, hypertension and obesity are highly associated with T2DM and metabolic syndrome. In our multivariate analysis, we included all these variables, but in the final model, the only metabolic factor independently associated with the risk of death and progression was the presence of T2DM. However, we cannot completely exclude the potential role of obesity and hypertension as confounders in our analysis. Patients with T2DM are characterized by insulin resistance, which results in high circulating insulin concentrations, and increased growth factors’ production. The stimulation of cell proliferation by hyperinsulinemia was first recognized in animal studies. Rats and mice with diabetes induced by streptozotocin or alloxan and characterized by hyperglycemia and insulin deficiency, display a longer latency period for cancer development, lower number of tumors, slower cancer progression, and smaller final tumor volume as compared with controls.^28^ Insulin and insuline-like growth factor-1 (IGF-1) generate their effects through insulin receptors and IGF-1 receptors, respectively, to promote cellular proliferation and inhibit apoptosis in many tissue types. These effects might contribute to cancer development.^29^ However, hyperglycemia may play a significant role in tumor progression, promoting DNA damage and causing the activation of different signaling pathways significantly associated with tumorigenesis and metastatization.^30^ Continuous exposure to high glycemic and insulin levels seems to stimulate cancer growth and progression, leading to a worse prognosis. Finally, the presence of renal and/or cardiac dysfunction, frequently observed in diabetic patients, can require dose reductions of cancer therapies and hence insufficient achievement of target drug concentrations.^31^

There are several limitations to this study. First, the data were collected retrospectively and reflect a single-institution experience. Second, this study included many decades (patients treated between 1979 and 2013), during which great advances have been made in the treatment of both kidney cancer and diabetes. Third, the lack of laboratory measurements is a limit; DM is a complicated disease that is characterized not only by hyperglycemia but also by other metabolic impairments (eg, levels of insulin or IGFs). Lastly, we could not analyze lifestyle variables related to glucose metabolism, such as physical activity and diet.

In conclusion, our data suggest that T2DM may be an independent negative predictor of survival in RCC patients. Information obtained from the present study may help to clarify cancer risks for patients with a history of T2DM. Moreover, an investigation of the relationship between T2DM and RCC has great significance from an epidemiological standpoint, to determine preventive measures and implement screening and follow-up strategies. However, the pathophysiology underlying cancer prognosis and diabetes remains uncertain and requires further investigation. A multi-institutional prospective study with a larger number of patients is needed to confirm these results.
